# Role of miRNA–mRNA Interactome in Pathophysiology of Arrhythmogenic Cardiomyopathy

**DOI:** 10.3390/biomedicines12081807

**Published:** 2024-08-09

**Authors:** Fernando Bonet, Oscar Campuzano, José Córdoba-Caballero, Mireia Alcalde, Georgia Sarquella-Brugada, Aitana Braza-Boïls, Ramon Brugada, Francisco Hernández-Torres, Maribel Quezada-Feijoo, Monica Ramos, Alipio Mangas, Juan A. G. Ranea, Rocío Toro

**Affiliations:** 1 Research Unit, Biomedical Research and Innovation Institute of Cadiz (INiBICA), Puerta del Mar University Hospital, 11009 Cádiz, Spain; fbonetmartinez@gmail.com (F.B.); josecordoba1995@gmail.com (J.C.-C.); alipio.mangas@uca.es (A.M.); 2 Medical Science Department, School of Medicine, University of Girona, 17003 Girona, Spain; georgia@brugada.org (G.S.-B.); rbrugada@idibgi.org (R.B.); 3 Institut d’Investigació Biomèdica de Girona (IDIBGI-CERCA), 17190 Salt, Spain; malcalde@gencardio.com; 4 Centro Investigación Biomédica en Red, Enfermedades Cardiovasculares (CIBERCV), 28029 Madrid, Spain; aitana_braza@iislafe.es; 5 Department of Molecular Biology and Biochemistry, University of Málaga, 29071 Málaga, Spain; ranea@uma.es; 6 Pediatric Arrhythmias, Inherited Cardiac Diseases and Sudden Death Unit, Cardiology Department, Sant Joan de Déu Hospital, 08950 Barcelona, Spain; 7 Arrítmies Pediàtriques, Cardiologia Genètica i Mort Sobtada, Malalties Cardiovasculars en el Desenvolupament, Institut de Recerca Sant Joan de Déu, 08950 Barcelona, Spain; 8 Cardiopatías Familiares, Muerte Súbita y Mecanismos de Enfermedad (CAFAMUSME) Research Group, Instituto de Investigación Sanitaria La Fe, 46026 Valencia, Spain; 9 Cardiology Service, Hospital Josep Trueta de Girona, 17007 Girona, Spain; 10 Medina Foundation, Technology Park of Health Sciences, 18016 Granada, Spain; fhtorres@ugr.es; 11 Department of Biochemistry and Molecular Biology III and Immunology, Faculty of Medicine, University of Granada, 18016 Granada, Spain; 12 Cardiology Department, Hospital Central de la Cruz Roja, 28003 Madrid, Spain; maribelquezada2000@gmail.com (M.Q.-F.);; 13 Medicine School, Alfonso X el Sabio University, 28007 Madrid, Spain; 14 Medicine Department, School of Medicine, University of Cadiz, 11003 Cádiz, Spain; 15 Lipid and Atherosclerotic Unit, Puerta del Mar University Hospital, 11009 Cadiz, Spain; 16 Institute of Biomedical Research in Málaga and platform of nanomedicine (IBIMA Plataforma BIONAND), 29071 Málaga, Spain; 17 Center for Biomedical Network Research on Rare Diseases (CIBERER), Instituto de Salud Carlos III (ISCIII), 28029 Madrid, Spain; 18 Spanish National Bioinformatics Institute (INB/ELIXIR-ES), Instituto de Salud Carlos III (ISCIII), 28020 Madrid, Spain

**Keywords:** arrhythmogenic cardiomyopathy, sudden cardiac death, RNA sequencing, microRNA, miRNA–mRNA

## Abstract

Arrhythmogenic cardiomyopathy is an inherited entity characterized by irregular cell–cell adhesion, cardiomyocyte death and fibro-fatty replacement of ventricular myocytes, leading to malignant ventricular arrythmias, contractile dysfunction and sudden cardiac death. Pathogenic variants in genes that encode desmosome are the predominant cause of arrhythmogenic cardiomyopathy. Moreover, signalling pathways such as Wnt/ß-catenin and transforming growth factor-β have been involved in the disease progression. However, still little is known about the molecular pathophysiological mechanisms that underlie arrhythmogenic cardiomyopathy pathogenesis. We used mRNA and small RNA sequencing to analyse the transcriptome of health and arrhythmogenic cardiomyopathy of autopsied human hearts. Our results showed 697 differentially expressed genes and eight differentially expressed miRNAs. Functional enrichment revealed mitochondrial respiratory-related pathways, impaired response to oxidative stress, apoptotic signalling pathways and inflammatory response-related and extracellular matrix response pathways. Furthermore, analysis of the miRNA–mRNA interactome identified eleven negatively correlated miRNA-target pairs for arrhythmogenic cardiomyopathy. Our finding revealed novel arrhythmogenic cardiomyopathy-related miRNAs with important regulatory function in disease pathogenesis, highlighting their value as potential key targets for therapeutic approaches.

## 1. Introduction

Arrhythmogenic cardiomyopathy (ACM) is a heterogeneous genetic entity characterized by ventricular arrhythmias, contractile dysfunctions and progressive fibro-adipose replacement of myocardium [1]. ACM encompasses arrhythmogenic right ventricular cardiomyopathy (ARVC), arrhythmogenic biventricular cardiomyopathy (ABVC) and arrhythmogenic left ventricular cardiomyopathy (ALVC), all predisposing the patient to ventricular electrical instability and sudden cardiac death (SCD) [2]. ACM is a rare condition with a prevalence of 1/5000–6.5/5000 (OMIM #107970; ORPHA247) that affects mainly young individuals and athletes. In 2020, the “Padua criteria” were proposed for the diagnosis of ACM but it has been recently improved [3]. Several features such as male sex, previous non-sustained ventricular tachyarrhythmias, extent of T-wave inversion, recurrent premature ectopic beats and lower biventricular ejection fraction are risk factors for subsequent malignant arrhythmogenic events [4].

Pathogenic variants in genes encoding desmosomal proteins, such as *PKP2, DSG2, DSC2* and *DSP*, constitute the main cause of ACM, although non-desmosomal forms of ACM also exist [5]. The inheritance of the disease is generally autosomic dominant; however, recessive forms with similar phenotypes have also been reported (*JUP* and *DSP* leading to Naxos and Carvajal diseases, respectively) [3]. All forms of ACM are characterized by low penetrance and variable expressivity, even in carriers of the same rare causative variant [6]. Genetic screening is crucial in ACM as it is one of the criteria on which the diagnosis of ACM is currently based [3].

The early identification of patients and adoption of personalized therapeutic measures remain the main current challenges. Hence, unravelling the pathophysiological mechanisms involved in the onset, progression and outcome of ACM will help with clinical diagnosis as well as risk stratification. From a pathophysiological perspective, hearts affected by ACM show a progressive loss of cardiomyocytes and fibro-fatty tissue replacement [7]. Although the pathological hallmark of the disease is usually present in the epicardial surface, septal and left ventricle areas can also show fibro-fatty infiltrations [8]. The desmosomes’ impairment affects the cell–cell junction, signalling at the intercalated discs and gaps union that functionally leads to intraventricular delay and re-entry circuits that provoke ventricular arrythmias. Furthermore, cardiomyocytes cannot cope with the mechanical strain, thus contributing to cardiomyocyte apoptosis.

Previous research focused on the comprehension of molecular mechanisms driving the ACM phenotype has demonstrated alterations of the Wnt/β-catenin pathway, which activates adipogenesis, fibrosis and apoptosis [9,10,11]. Another dysregulated pathway in ACM is the Hippo pathway, which responds to mechanical stimuli or cell–cell interactions regulating cell proliferation, apoptosis and cell fate. Moreover, activation of the Hippo pathway might inhibit the canonical Wnt signalling, thus promoting adipogenesis [12,13]. MicroRNAs (miRNAs) are small non-coding RNAs with a crucial role in the physiological development of the heart and cardiovascular diseases [14]. More recently, miRNA have also been shown to play an important role in ACM [11,15]. In the present work, we used mRNA and miRNA sequencing to analyse the transcriptome in ACM and healthy hearts.

## 2. Materials and Methods

### 2.1. Study Population

We recruited four frozen right ventricle (RV) myocardial biopsies from ACM patients (A1.R, A2.R, A4.R and A6.R) and four RV myocardial samples from unrelated ACM subjects (B1.R, B2.R, B3.R and B5.R) who died from conditions other than cardiac diseases as control group (Table 1). In ACM samples, pathogenic variant in each case is showed in Table 1. In postmortem analysis, all samples showed less than 60% of residual myocytes, with fibrosis substitution in right ventricular myocardium free wall, some of them even with fat-ty replacement. In addition, family history (first-degree relative) of ACM was confirmed in all cases. The criteria to collect samples was a definite clinical diagnosis of ACM. The second step was to include samples carrying genetic variants with conclusive deleterious role, all in genes currently associated with ACM.

### 2.2. Ethics

This study was approved by the appropriate ethics committees. The ethical research principles were fulfilled following the Helsinki Declaration, and further amendments, and the Belmont report. This study also adhered to two legal provisions governing human research and the Spanish Organic Law 15/1999 for the Regulation of Automated Processing of Personal Data. All procedures performed were in accordance with the ethical standards of the institutional research committee and was approved by the local Ethics Committee (CEIM: 2021.185, CEIC 1315-N-21, 2018/0417 and 2014/0306).

### 2.3. Myocardial Tissue Collection

This is a multicentre study that involved Research Biomedicine Institute Josep Trueta (IDIBGI), Girona; Health Research Institute La Fe, Hospital La Fe of Valencia, Valencia; and Research Institute of Biomedicine and Innovation (INIBICA), Cádiz. Heart samples from human autopsies were stored at −80 °C until they were analysed.

### 2.4. RNA Extraction

The TRI Reagent (Sigma-Aldrich, St Louis, MO, USA) was used to isolate and purify total RNA according to the manufacturer’s instructions. Then, DNase treatment was performed using RNA clean and concentrator-5 kit (Zymo Research, Irvine, CA, USA). RNA was quantified using a Qubit RNA High-Sensitivity Assay kit in the Qubit^®^ 2.0 Fluorometer (Life Technologies, Carlsbad, CA, USA). The quality and integrity of total RNA were controlled on the Agilent Technologies 2100 Bioanalyzer (Agilent Technologies, Santa Clara, CA, USA).

### 2.5. RNA-Sequencing Analysis and Bioinformatics

Standard specific mRNA-sequencing (mRNA-seq) libraries were generated using the NEBNext Ultra II Directional RNA Library Prep Kit for Illumina using the NEBNext Poly(A) mRNA Magnetic Isolation Module (New England Biolabs, Ipswich, MA), and single-end libraries were sequenced on an Illumina SE100 Platform with an output of ~680M reads per sample. Standard miRNA libraries were generated using the NEXTFLEX small RNA-seq kit v3 (Perkin Elmer, Waltham, MA, USA), and single-end libraries were sequenced on an Illumina SE60 Platform with an output of ~2 M reads per sample.

For trimming and aligning raw data, fastq sequence reads were uploaded to the European version of the Galaxy platform [16]. Reads were trimmed with the Trim Galore software (Galaxy Version 0.6.7 + galaxy0) and aligned to the built-in human reference genome December 2013 (GRCh38/hg38) with the RNA STAR Gapped-read mapper (Galaxy Version 2.7.10b + galaxy3). For gene expression analyses, bam files were downloaded from the Galaxy server and further analysed with the different RStudio packages downloaded from the Bioconductor website (http://bioconductor.org, accessed on 8 July 2023). Reads were assigned to genes by means of the “featureCounts” function of the “Rsubread” package, version 2.10.5 [17], and annotation files human release 43 (GRCh38.p13) (https://www.gencodegenes.org/human/release_43.html, accessed on 18 April 2024) and Chromosomal coordinates of *Homo sapiens* microRNAs (https://www.mirbase.org, accessed on 18 April 2024) were used for mRNA and miRNA analysis, respectively. Only the mapped reads were used to calculate gene expressions. The library size of each experimental point ranged from 37,342,862 to 51,420,486 sequences and from 318,097 to 1,898,024 sequences for mRNA and miRNA analysis, respectively.

The difference in the library size ranges between mRNA and miRNA is attributed to the inherent characteristics of the RNA types and their respective sequencing techniques. mRNA, being longer and more varied compared to miRNA, requires a greater number of reads to ensure adequate coverage and precise quantification of gene expression. In contrast, miRNA, which is significantly shorter, needs fewer reads to achieve comprehensive representation. These differing sequencing requirements account for the observed variations in library sizes between mRNA and miRNA.

The differential gene expression analyses were performed with package ‘DESeq2’ version 1.36.0 [17]. All the gene comparisons with a *p*-value <0.05 and a fold-change >2 were considered differentially expressed under the experimental conditions. Functional Over Representation Analysis (ORA) based on Kyoto Encyclopedia of Genes and Genomes (KEGG) and Gene Ontology (GO) were conducted with the package ‘clusterProfiler’ version 4.4.4. [18]. The gene sets with a *p*-value <0.05 were considered overrepresented under the experimental conditions. Finally, to identify miRNA–mRNA interaction, we constructed a pipeline using the DEMs and DEGs between ACM and control cohorts. We used Diana-microT, ElMMo, Microcosm, Miranda, Mirdb, Pictar, PITA and Targetscan prediction tools to select mRNA targets.

### 2.6. Statistical Analysis

Data are expressed as mean ± SEM, and n denotes the number of replicates for each experiment. Outliers were identified through the Rout method, using Q = 1%. The normal distribution of each variable was verified with the Shapiro–Wilk test. Statistical differences (*p* < 0.05) between the experimental groups were assessed using a two-tailed, unpaired Student’s *t* test for Gaussian distributions. For non-Gaussian distributions, a Mann–Whitney non-parametric test was used. All the statistical analyses were performed using GraphPad Prism 9.0 software (San Diego, CA, USA).

## 3. Results

### 3.1. ACM and Controls mRNA and miRNA Expression Profiles

**Figure 1:**
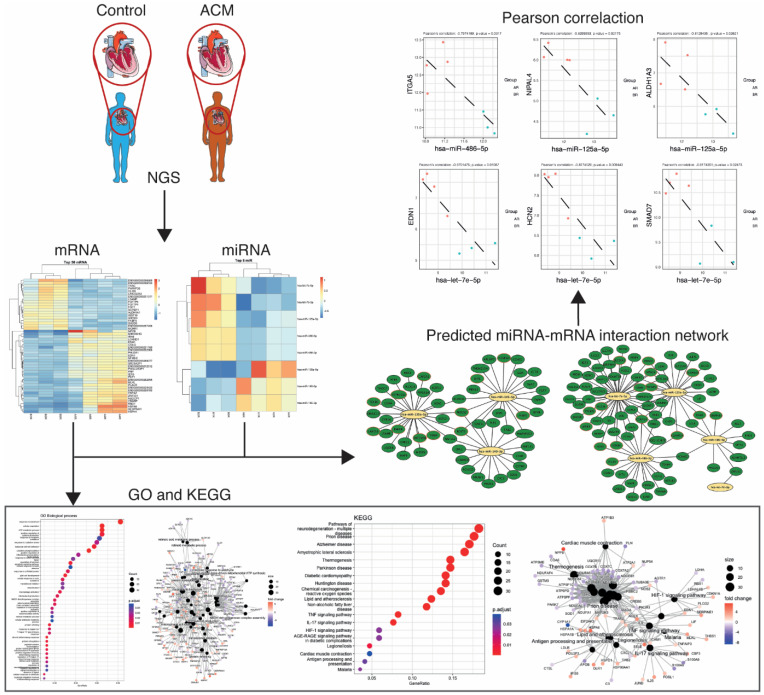
A flowchart of the study design.

**Figure 2:**
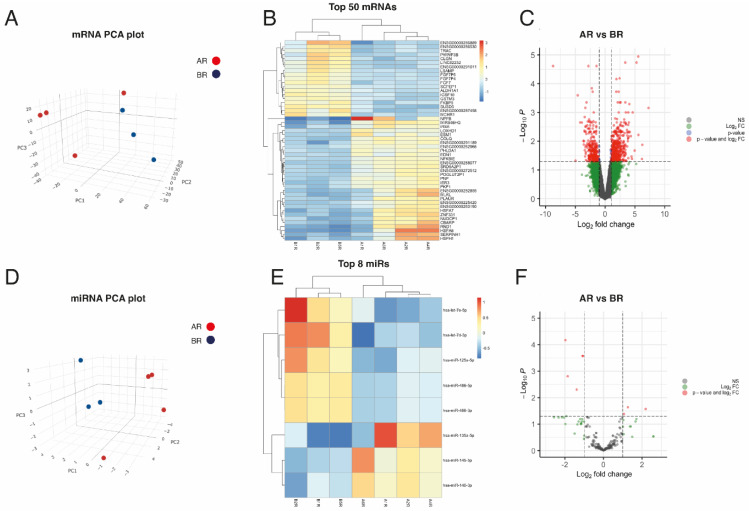
Exploratory analysis of paired miRNA and mRNA expression in heart samples. (**A**) 3D-Principal Components Analysis plot, based on correlation matrix, for mRNA expression in ACM (n = 4) and control (n = 3) tissue samples. (**B**) Heatmap of the top 50 most DEMs sorted by absolute FC (all of them having FDR < 0.05). (**C**) Volcano plot of the mRNAs, highlighting in grey those not statistically significant with FDR > 0.05 and absolute FC < 2 (abslog2FC < 1); in blue, those with FDR < 0.05 but absolute FC < 2 (abslog2FC < 1); in green, those with absolute FC > 2 (abslog2FC > 1) but FDR > 0.05; and in red, those with FDR < 0.05 and absolute FC > 2 (abslog2FC > 1). (**D**) 3D-Principal Components Analysis plot, based on correlation matrix, for miRNA expression in ACM (n = 4) and control (n = 3) tissue samples. (**E**) Heatmap of the top 8 most DEMs sorted by absolute FC (all of them having FDR < 0.05). (**F**) Volcano plot of the miRNAs, highlighting in grey those not statistically significant with FDR > 0.05 and absolute FC < 2 (abslog2FC < 1); in green, those with absolute FC > 2 (abslog2FC > 1) but FDR > 0.05; and in red, those with FDR <0.05 and absolute FC > 2 (abslog2FC > 1).

We performed RNA-seq to investigate whether differences exist in the mRNA expression profiles of the myocardium of ACM and control patients. The genetic data of the 8 patients included in this study are shown in Table 1. The filtered RNA-seq read-set identified ≈ 29,568 genes. We used principal component analysis (PCA) to visualize the sample clustering for the most variably expressed genes (Figure 1). No outliers were observed in the PCA (Figure 2A).

In total, 689 genes were differentially expressed between ACM and controls, with a Log_2_ fold-change (FC) > 0.5 using a 5% false discovery rate (FDR). From those genes, 383 were upregulated and 306 were downregulated in the ACM samples (Figure 2B,C). We also analysed the miRNA expression profile by way of small RNA-seq to investigate the differential expression between ACM and the control groups. According to the PCA, the miRNA expression profile of the ACM cohort differed significantly from that of the control sample’s autopsies and no outliers were observed (Figure 2D). After RNA-seq analysis, 290 miRNAs were detected in all heart samples. The most highly expressed miRNA in all myocardial samples was miR-1-3p (Table S1). We identified eight DEMs between the ACM and control heart samples (Log_2_ FC > 0.5; FDR ≤ 0.05). Three of them (miR-135a-5p, miR-140-3p and miR-145-5p) were upregulated, and five (miR-486-5p, miR-486-3p, miR-125a-5p, let-7e-5p and let-7d-3p) were downregulated in ACM hearts as compared to the controls (Figure 2E,F).

**Figure 3:**
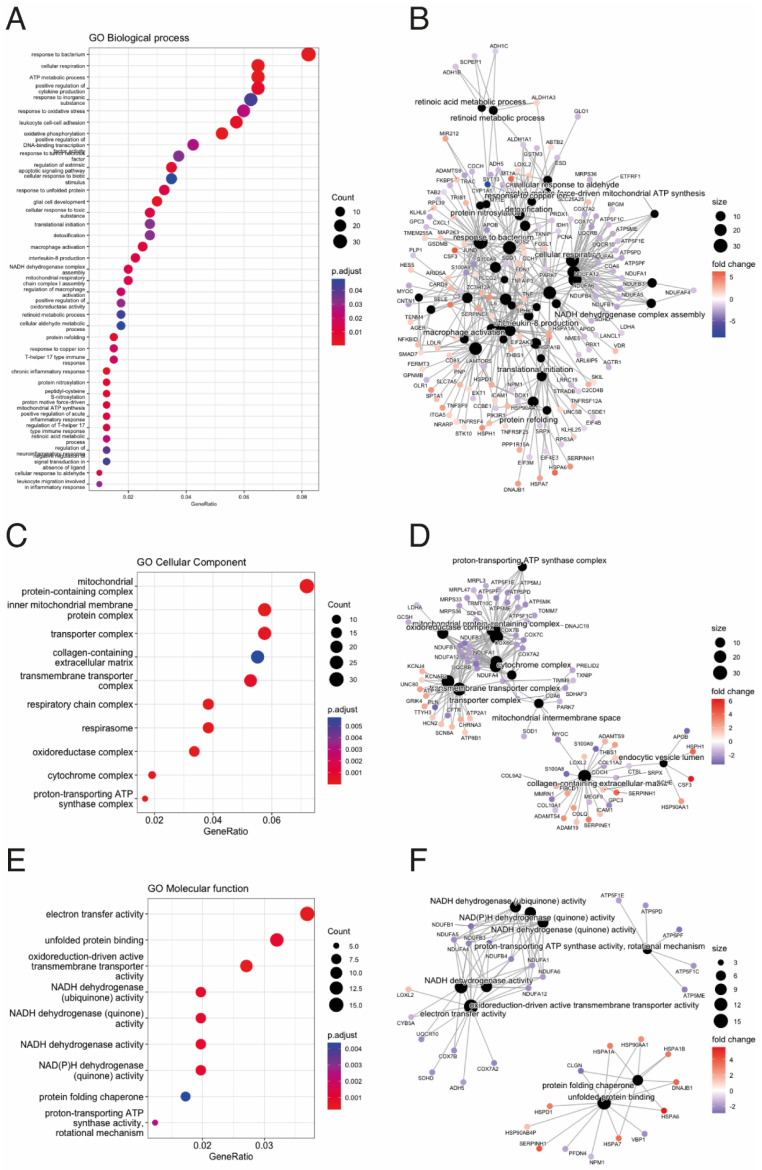
Gene Ontology (GO) Over Representation Analysis (ORA) between ACM and control groups. (**A**) Dot plot for Biological Process (BP) analysis. (**B**) Gene-Concept Network plot showing the linkages of differential expressed genes and BP terms. (**C**) Dot plot for Cellular Component (CC) analysis. (**D**) Gene-Concept Network plot showing the linkages of differential expressed genes and CC terms. (**E**) Dot plot for Molecular Function (MF) analysis. (**F**) Gene-Concept Network plot showing the linkages of differential expressed genes and MF terms.

**Figure 4:**
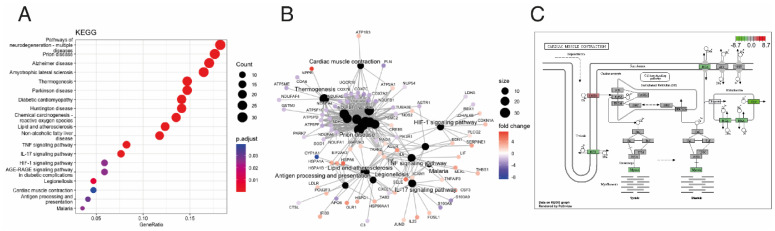
Kyoto Encyclopedia of Genes and Genomes (KEGG) Over Representation Analysis (ORA) between ACM and control groups. (**A**) Dot plot for KEGG analysis. (**B**) Gene-Concept Network plot showing the linkages of differential expressed genes and KEGG pathways. (**C**) Down-regulated “cardiac muscle contraction” KEGG pathway; gene expression values are mapped to gradient.

To identify the biological mechanisms related to ACM disease, we performed pathway enrichment on the DEGs in our study. According to the number of counts and categorized by the biological process (BP), we found mitochondrial respiratory-related pathways, response to oxidative stress, apoptotic signalling pathways, inflammatory response-related pathways and retinoic acid metabolic processes to be among the most enriched GO terms (Figure 3A,B). In the cellular component (CC), mitochondrial respiratory- and extracellular matrix-related pathways were among the most enriched pathways in ACM (Figure 3C,D). Similarly, in terms of molecular function (MF), mitochondrial respiratory- and ER stress-related pathways were among the most enriched pathways in ACM (Figure 3E,F). Finally, KEGG pathway analysis showed that these DEGs were mainly involved in diabetic cardiomyopathy, lipid and atherosclerosis, inflammation response-related pathways and cardiac muscle contraction (Figure 4A–C).

### 3.2. Integrative Analysis Identified miRNA–mRNA Interaction for ACM

**Figure 5:**
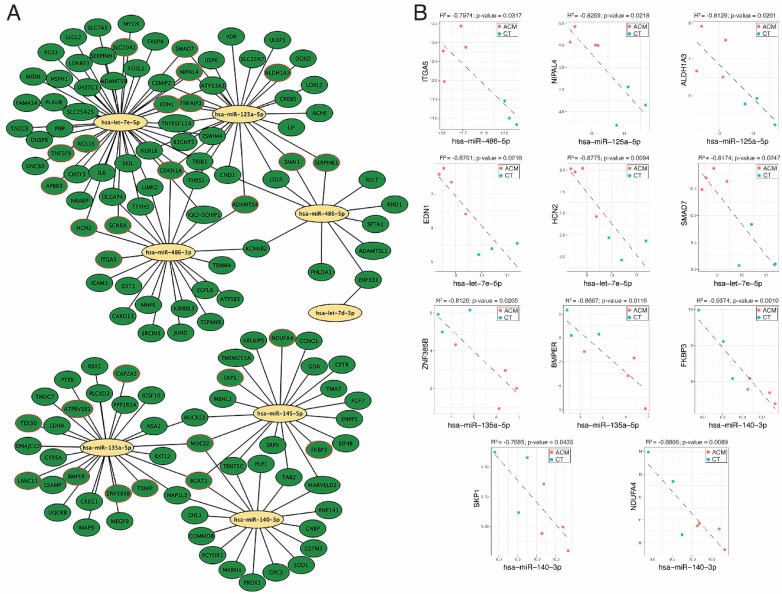
miRNA–mRNA interaction analysis. (**A**) Network of selected miRNA–mRNA interactions. (**B**) Negatively correlated miRNA–mRNA pairs predicted simultaneously, at least in three databases used in our pipeline.

Considering that mRNAs are typically targeted by many miRNAs and each miRNA targets multiple mRNAs, we established an effective pipeline to identify the miRNA–mRNA interaction for ACM using the DEMs and DEGs between ACM and controls. We used Diana-microT, ElMMo, Microcosm, Miranda, Mirdb, Pictar, PITA and Targetscan prediction tools to select the mRNA targets. We only selected negatively correlated miRNA–mRNA pairs. This prediction and filtering approach resulted in 304 pairs comprising eight miRNAs and their 135 likely target mRNAs (Figure 5A and Tables S1–S3). The list of miRNA–gene pairs includes already validated pairs using the Mirtarbase and Tarbase databases (Figure 5A and Tables S1–S3).

Among all miRNA–mRNA pairs, we selected those genes meeting the following criteria: (1) genes predicted as the target of a DEM by at least three miRNA target prediction tools, (2) target genes that were experimentally validated and stored in a database such as the Mirtarbase and/or Tarbase databases and (3) target genes shared by at least two DEMs (Table 2). Pearson correlation tests were performed to identify the miRNA-target pairs. Our miRNA–mRNA analysis identified 11 negatively correlated miRNA-target pairs for ACM: miR-486-5p/ITGA5, miR-125a-5p/NIPAL4, miR-125a-5p/ALDH1A3, let-7e-5p/EDN1, let-7e-5p/HCN2, let-7e-5p/SMAD7, miR-135a-5p/ZNF385B, miR-135a-5p/BMPER, miR-140-3p/FKBP3, miR-140-3p/SKP1 and miR-140-3p/NDUFA4 (R < −0.7, *p*-value < 0.05) (Figure 5B).

## 4. Discussion

In the present study, we performed for the first time a wide-ranging characterization of the mRNA and miRNA transcriptome of myocardial tissue from ACM patients. We generated an ACM-specific miRNA–target transcript interaction network, thereby providing the first unbiased analysis of miRNAs and their targets in the context of ACM. Hereto, we identified 689 mRNAs and 53 miRNAs with significant differential expression in ACM. Subsequent pathway enrichment analysis of the DEGs showed significant enrichment for genes, mainly regarding mitochondrial respiration, extracellular matrix, oxidative stress, ER stress, apoptosis, cell–cell adhesion, inflammation, retinoic acid metabolic processes, diabetic cardiomyopathy, lipid and atherosclerosis and cardiac muscle contraction, among others. We created an ACM-associated miRNA interactome of eight miRNAs and their 135 likely target mRNAs.

ACM is characterized by the death of cardiomyocytes followed by inflammation and the progressive accumulation of fibro-fatty tissue. Alterations in Wnt/ß-catenin and the Hippo pathway due to abnormal cell–cell adhesion and intracellular signalling, caused by deleterious rare variants located in genes encoding desmosomal proteins, is considered the main cause [10,12]. This is in consonance with the deregulation of genes involved in cell–cell adhesion, extracellular matrix-related pathways, apoptosis and cardiac muscle contraction. Our results showed dysregulation of cardiac inflammation and lipid transport pathways consistent with another research. Rainer et al. analysed the coding and non-coding transcriptome of human cardiac stromal cells derived from endomyocardial biopsies of ACM patients. They found that, in addition to cell–cell adhesion, deregulated genes were also involved in cardiac inflammation and lipid transport [19]. Similarly, RNA-seq data from left ventricular tissue from deceased ACM patients showed dysregulation of cell–cell adhesion, extracellular matrix, and inflammation [20]. In alignment with these findings, Lin et al. (2023) profiled the region-resolved transcriptome and proteome of healthy and dilated cardiomyopathy (DCM) human myocardial tissue and obtained an extensive dataset. Based on the core proteome and transcriptome characteristics of healthy hearts, chamber-specific proteome alterations were further revealed in end-stage DCM, among which extracellular matrix (ECM), mitochondrial function, and muscle contraction were the most dysregulated biological processes. These results reinforce the relevance of ECM and mitochondrial dysfunction in cardiomyopathies and provide additional context for our findings in ACM, highlighting the broader implications of these dysregulated processes across different types of cardiomyopathies [21].

The mitochondrial impairment might constitute substrates for electrical and structural remodelling in ACM hearts, playing, therefore, a role in the electrical stability [22]. Lippi et al. recently demonstrated epigenetic and gene expression profiles of cardiac mesenchymal stromal cells and confirmed for the first time that mitochondrial dysfunction is present in ACM [23]. This group also proposed that oxidative stress represents a cofactor contributing to the pathogenesis of ACM [24], which might be a consequence of mitochondrial dysfunction. This fact was evidenced in patients carrying pathogenic *PKP2* variants [25]. Consistently, our results revealed dysregulation of mitochondrial respiration pathways and oxidative stress-related pathways. Similarly, we found that DEGs in ACM are involved in ER stress pathways, which is in line with previous observations showing the overexpression of ER stress markers in the myocardial tissue of ACM murine models [26]. A comparative analysis of pathways identified in this study versus the reported literature is shown in Table 3.

We identified eleven negatively correlated miRNA-target transcript pairs: miR-486-5p/ITGA5, miR-125a-5p/NIPAL4, miR-125a-5p/ALDH1A3, let-7e-5p/EDN1, let-7e-5p/HCN2, let-7e-5p/SMAD7, miR-135a-5p/ZNF385B, miR-135a-5p/BMPER, miR-140-3p/FKBP3, miR-140-3p/SKP1 and miR-140-3p/NDUFA4. The epicardium is a source of multiple cardiac cell types, including fibroblasts, playing a key role during cardiac development and remodelling through the epithelial-to-mesenchymal (EMT) process [36,37,38,39]. Several studies have proposed the epicardium as the initial site of manifestation of classic ACM [40,41,42,43,44]. Integrin alpha 5 (ITGA5) is necessary for proper heart morphogenesis, and the binding of ITGA5 to the epicardial secreted fibronectin (FN1) is required for cardiomyocyte maturation [45,46,47,48]. In the adult heart, ITGA5 has been found to be upregulated in the atrial tissues of patients with atrial fibrillation, whereas epicardial secreted fibronectin has been identified as a source of fibroblasts through the EMT process [32]. Moreover, ITGA5 has been suggested as a promotor of adipocyte fibrosis-related gene expression [49]. Although in distinct biological contexts both ITGA5 and miR-486-5p have been reported to modulate the TGF-β-mediated EMT process, adipogenesis and fibrosis [35,49,50,51,52,53], the miR-486-5p/ITGA5 axis has been suggested as a potential target to modulate fibro-fatty infiltration of subepicardial layers into the myocardium. Interestingly, SMAD7, a negative regulator of TGF-ß signalling [54], was also deregulated in ACM samples, supporting the notion that TGF-ß signalling might be a key regulator of epicardial EMT processes in ACM. In colorectal cancer (CRC), TGF-β1 induces a partial EMT (pEMT) process and collective cell invasion without full mesenchymal transition, maintained by Smad transcription factors. This mechanism might similarly induce EMT processes in epicardial cells in ACM, leading to fibroblast accumulation [51]. miR-486-5p also plays a role in aging and adipogenesis in human adipose tissue-derived mesenchymal stem cells (hAT-MSCs) by targeting SIRT1, inhibiting proliferation and differentiation. This suggests miR-486-5p might similarly affect epicardial adipogenesis in ACM, contributing to its fibro-fatty phenotype [53]. ITGA5 plays a crucial role in adipocyte differentiation, where its reduction promotes adipogenesis and overexpression inhibits it. This regulatory role of ITGA5 could be disrupted in ACM, leading to pathological adipocyte accumulation [50]. Additionally, miR-486-5p inhibits adipogenesis in mesenchymal stem cells and prevents steroid-induced osteonecrosis by targeting TBX2 and upregulating p21. These anti-adipogenic effects could be leveraged to counteract ACM’s adipogenic component. Thus, the miR-486-5p/ITGA5 axis is a critical regulatory pathway in ACM, influencing both fibroblast and adipocyte behaviour. miR-486-5p has also been identified as a tumour suppressor in non-small cell lung cancer (NSCLC) progression, where it modulates TGF-β signalling and EMT processes. High expression of SMAD2, a downstream effector of TGF-β, correlates with poor prognosis in NSCLC and promotes EMT processes. miR-486-5p targets SMAD2, inhibiting TGF-β-induced EMT processes and metastasis in NSCLC cells [51]. Similarly, in ACM, miR-486-5p could inhibit TGF-β-mediated EMT processes by targeting ITGA5, thus preventing fibro-fatty infiltration. This highlights miR-486-5p as a potential modulator of epicardial EMT processes in ACM, which may be crucial for controlling disease progression. Understanding the miR-486-5p/ITGA5 axis offers potential therapeutic insights. Therapeutically, modulating this pathway could inhibit pathological EMT processes and adipogenesis, reducing fibro-fatty infiltration in ACM. Although little is known about the role of let-7e-5p in cardiac disease, several studies suggest an active role in the pathogenesis of heart failure [55,56]; however, its role in regulating TGF-ß signalling remains to be investigated.

Bone morphogenetic proteins (BMPs) are members of the TGF-ß superfamily. Several studies have described BMPER as a regulator of BMP signalling [57,58,59]. Besides its regulatory function in endothelial biology [57,60,61,62,63], BMPER has also been identified as an EMT process-related gene [64]. Furthermore, BMPER has been proposed as a potential regulator of epicardial adipose tissue (EAT) mesenchymal stem cells (MSCs). EAT is an ectopic visceral fat deposit enveloping the heart that acts as an active endocrine organ with effects on myocardial metabolism [65,66]. Curiously, the amount of right and left ventricular EAT has been associated with ACM and the disease’s severity in humans [27]. Hence, BMPER may play a role in ACM regulating EAT stem cells. Interestingly, miR-135a-5p has been reported to modulate adipogenesis in EAT MSCs through the Hippo signalling pathway [28,34]. This miRNA is downregulated during preadipocyte differentiation, and its overexpression impairs adipogenic marker gene expression, emphasizing its regulatory role in adipogenesis [27]. Furthermore, miR-135a-5p influences the canonical Wnt/β-catenin signalling pathway by targeting APC, thereby affecting cell differentiation processes [28]. In the context of ACM, both BMPER and miR-135a-5p are relevant due to their roles in regulating adipogenesis and fibrosis. BMPER has been shown to regulate BMP2-mediated signalling, influencing EMT processes and extracellular matrix deposition [63]. Additionally, miR-135a-5p has been reported to modulate adipogenesis in EAT MSCs through the Hippo signalling pathway, and its dysregulation may contribute to pathological changes in ACM [28]. The connection between BMPER and miR-135a-5p in regulating EAT MSCs presents a novel perspective on ACM treatment. The miR-135a-5p/BMPER axis may offer new therapeutic opportunities by targeting key pathways involved in fibrosis and adipogenesis. Given the association between EAT volume and ACM severity, modulating this axis could potentially mitigate fibro-fatty infiltration and improve disease outcomes.

ALDH1A3 is a member of the Aldehyde dehydrogenase 1 family (ALDH1) that participates in the retinoic acid (RA) biosynthesis [67], a vitamin A metabolite that plays a significant role in the contribution of the epicardium to heart development and function [68]. In the mouse heart, RA has been described to mediate dilated cardiomyopathy (DCM) and cardiomyocyte apoptosis after myocardial infarction, suggesting a role in the adult human heart as well [33,69]. Interestingly, RA has been implicated in adipogenesis through transcriptional regulation of PPARγ [70]. In this sense, the induction of PPARγ expression mediated by RA can convert both primary myogenic cells and the myoblast cell line to adipogenic cells in avian samples [71]. Our data showed miR-125a-5p as a potential target of ALDH1A3. Curiously, it has been shown that inhibition of this miRNA accelerates 3T3-L1 preadipocyte differentiation and upregulation of fatty acid metabolism-related genes [30]. Accordingly, we observed downregulation of miR-125a-5p and upregulation of ALDH1A3 in ACM samples. The ALDH1A3/miR-125a-5p axis therefore offers a novel perspective on the regulation of adipogenesis in ACM. ALDH1A3-mediated RA synthesis and subsequent RA-induced PPARγ expression could drive adipogenic differentiation in the epicardium, contributing to the fibro-fatty remodelling characteristic of ACM. The downregulation of miR-125a-5p, which typically inhibits adipogenesis, further supports this process, suggesting a coordinated regulatory mechanism involving both ALDH1A3 and miR-125a-5p. Therapeutically, targeting the ALDH1A3/miR-125a-5p axis could modulate adipogenesis and fibrosis in ACM, potentially mitigating disease progression. However, further research is needed to validate these findings and explore the clinical applications of manipulating the ALDH1A3/miR-125a-5p axis in ACM.

Altogether, our results reinforce the concept of the epicardial contribution to fibro-fatty remodelling in ACM and offer potential new therapeutic targets for the treatment of the disease. HCN2 is one of a family of four genes (HCN1-4) that encodes a hyperpolarization-activated, cyclic nucleotide-gated cation channel [31] whose altered function is associated with arrhythmogenic events [72]. Although alteration in the expression of HCN2 has not been related to arrhythmias in physiological conditions, under pathological conditions HCN2 overexpression induces arrhythmias [73,74], which is consistent with higher expression levels of HCN2, therefore suggesting that HCN2 might play a role in ACM. Dysregulation of Endothelin-1 (EDN1) has been linked to DCM [72]. Furthermore, EDN1 expression has been positively correlated with the levels of superoxide (SOD1 and SOD2) and the amount of collagen in the mouse heart [75]. Accordingly, we observed upregulation of EDN1 in ACM hearts, suggesting EDN1 as an important player in controlling fibrosis and oxidative stress in ACM.

Regarding the miRNA–mRNA pairs, miR-125a-5p/NIPAL4, miR-135a-5p/ZNF385B, miR-140-3p/FKBP3, miR-140-3p/SKP1 and miR-140-3p/NDUFA4, very little or nothing is known about the function of these genes in cardiac disease. Whereas Skp1 has been related to cardiac hypertrophy and the degradation of key sarcomeric proteins [29,76], NDUFA4 has been associated with cardiomyocyte apoptosis and mitochondrial dysfunction, two ACM-related processes [77]. Further research will be required to explore the role of NIPAL4, ZNF385B and FKBP3 in ACM. Finally, miR-135a-5p and miR-140-3p have been involved in a wide range of cardiac-related processes, such as cardiomyocyte apoptosis, cardiomyocyte hypoxia, oxidative stress, fibrosis, cardiac inflammation, cardiac hypertrophy and heart failure, making them good candidates for therapeutic targets for ACM.

Targeting miRNAs in preclinical and clinical settings shows promise for treating various diseases, including cancer. Preclinical studies demonstrate the potential of miRNA modulation in altering disease progression and overcoming resistance. Advanced techniques like CRISPR and next-generation sequencing enhance our understanding of miRNA dysregulation. However, challenges such as toxicity and off-target effects need to be addressed for successful clinical translation. Ongoing clinical trials are evaluating miRNA-based therapies, emphasizing the importance of ensuring safety and efficacy in clinical applications. Continued research is crucial to harness the therapeutic potential of targeting miRNAs and non-coding RNAs for improved patient outcomes [78].

In our study, there are some limitations to mention. First is the number of samples. It is difficult to obtain human ventricular samples in proper condition to perform analyses such as those we performed in our study. However, we have a set of ACM samples which underwent clinical diagnostics and the gene variant was determined as the definite cause of disease; this is one of the main points nowadays as a large portion of rare variants remain with no definitive causality. Another limitation is the confirmation/validation of all these interactions, despite some of them having been previously validated. Moreover, it is important to take into account that the heart samples came from deceased individuals and RNA integrity could be affected by this condition. It is because of this that our control group shares similar postmortem intervals.

In summary, the evaluation of these miRNA–mRNA interactions highlight the potential for developing therapeutic agents that target these miRNAs as a promising approach in treating ACM. Therefore, this study successfully identified key miRNAs associated with ACM, offering new perspectives on the underlying mechanisms of ACM’s development.

## Figures and Tables

**Table 1:** Genetic data of samples with disease confirmed and pathogenic variant identified. ACM: Arrhythmogenic Cardiomyopathy; LP: Likely Pathogenic; NA: Not Available; P: Pathogenic; RV: Right Ventricle; VUS: Variant of Unknown Significance.

Sample	Phenotype	Gene	Protein	Nucleotide	dbSNP	gnomAD	ClinVar	ACMG
A1.R	ACM	*FLNC*	p.Arg1370Ter	c.4108C > T	rs1342121466	4/1450766 (0.0002%)	P	P
A2.R	ACM	*PKP2* *DSG2*	p.Lys678ArgfsTer12 -	c.1881del c.523 + 2dup	rs764817683 rs2073126642	NA 2/1451740 (0.0001%)	P VUS	P LP
A4.R	ACM	*PKP2*	p.Arg79Ter	c.235C > T	rs121434420	22/1577370 (0.001%)	LP	LP
A6.R	ACM	*TMEM43*	p.Ser358Leu	c.1073C > T	rs63750743	2/1461886 (0.0001%)	P	P
B1.R	Control	-	-	-	-	-	-	-
B2.R	Control	-	-	-	-	-	-	-
B3.R	Control	-	-	-	-	-	-	-
B5.R	Control	-	-	-	-	-	-	-

**Table 2:** Main miRNA–gene interactions predicted.

Counts							
Sample A1R	Sample A2R	Sample A4R	Sample A6R	Sample B1R	Sample B2R	Sample B5R	Gene Symbol
268,147	333,296	319,027	39,769	149,975	223,451	826,406	hsa-miR-1-3p
110,587	91,623	77,600	49,265	38,476	47,353	180,699	hsa-miR-143-3p
26,647	70,761	71,180	51,234	63,687	81,431	160,503	hsa-let-7a-5p
27,074	45,652	39,339	18,103	25,298	41,027	111,000	hsa-let-7f-5p
25,882	52,083	43,790	25,771	21,360	25,545	85,002	hsa-miR-26a-5p
28,171	45,954	34,588	8582	17,237	12,243	56,518	hsa-miR-30d-5p
13,406	53,514	23,570	6424	8590	6284	34,382	hsa-miR-133a-3p
17,567	26,356	25,662	9669	11,579	12,041	39,905	hsa-miR-24-3p
17,871	16,799	14,542	4624	5552	4409	25,811	hsa-miR-30a-5p
10,120	14,886	12,774	4398	5066	5808	20,783	hsa-miR-126-3p
8811	13,206	12,865	4841	5804	6031	20,016	hsa-miR-3074-5p
4907	18,329	10,663	3449	6000	6092	18,876	hsa-miR-30c-5p
4371	12,644	10,991	5905	5633	5314	18,177	hsa-miR-125b-5p
4524	11,010	10,114	5501	6172	6933	18,603	hsa-let-7g-5p
6351	9710	7213	3066	3287	5284	15,622	hsa-miR-27b-3p
2106	7043	6079	2008	5542	7547	16,297	hsa-miR-125a-5p
3642	10,930	6973	2556	3627	4263	12,207	hsa-miR-23b-3p
4042	10,743	6644	1492	2689	2598	10,319	hsa-miR-378a-3p
2029	6048	4382	2687	4514	3126	9480	hsa-miR-92a-3p
2838	7362	5436	3769	2454	2178	6331	hsa-miR-23a-3p
3568	4394	3894	2565	3678	2126	9118	hsa-miR-16-5p
5040	4232	4240	1244	2229	2111	8984	hsa-miR-22-3p
2482	5222	4175	2261	1938	3021	8180	hsa-miR-26b-5p
2953	4191	2876	2977	4368	1119	7761	hsa-miR-451a
1496	3889	2983	1273	2556	2826	10,222	hsa-miR-486-5p
1496	3889	2983	1273	2556	2826	10,222	hsa-miR-486-3p
1872	3873	2903	2441	2029	2386	8070	hsa-let-7i-5p
1796	4135	3928	3380	1426	1829	7013	hsa-miR-199a-3p
817	2593	2470	3058	2414	3316	6456	hsa-let-7c-5p
3914	3111	3522	2173	1043	1457	5324	hsa-miR-21-5p
1840	3173	2807	1327	1658	1086	4991	hsa-miR-103a-3p
1840	3173	2807	1327	1658	1086	4991	hsa-miR-103b
2528	2397	2317	272	1096	1841	6253	hsa-miR-499b-3p
2528	2397	2317	272	1096	1840	6253	hsa-miR-499a-5p
1664	3486	2368	1272	1048	1185	5165	hsa-miR-99b-5p
1518	3079	2508	2021	848	946	4498	hsa-miR-99a-5p
2306	2956	2142	874	1256	1034	4838	hsa-miR-181a-5p
764	2217	1593	2737	1488	1875	4178	hsa-let-7b-5p
3068	2358	2571	523	976	664	4526	hsa-miR-30e-5p
1377	3945	2391	2752	671	508	2690	hsa-miR-145-5p
894	2055	1956	1684	712	909	3489	hsa-miR-199b-3p
1378	2869	1863	519	888	720	3044	hsa-miR-30e-3p
927	1861	1642	873	897	963	3769	hsa-miR-423-3p
927	1861	1642	873	897	963	3769	hsa-miR-3184-5p
912	1552	1243	2236	719	516	2638	hsa-miR-100-5p
951	2629	1462	1280	536	278	1718	hsa-miR-140-3p
1289	1841	1288	706	619	593	1990	hsa-miR-27a-3p
928	1654	1220	515	378	367	1917	hsa-miR-151a-3p
729	1841	1080	386	513	522	1785	hsa-miR-30a-3p
1095	1195	1101	748	560	487	1617	hsa-miR-29a-3p
913	1935	1215	466	387	249	1449	hsa-miR-30b-5p
603	1136	945	598	764	562	1959	hsa-miR-191-5p
267	588	593	469	840	1560	2217	hsa-let-7e-5p
543	1081	910	461	539	605	1712	hsa-miR-151a-5p
71	203	353	759	862	416	2886	hsa-miR-10a-5p
267	768	737	486	849	777	1490	hsa-let-7d-5p
431	821	735	377	481	704	1800	hsa-miR-98-5p
96	163	236	1239	872	287	1942	hsa-miR-10b-5p
495	983	831	383	366	320	1333	hsa-miR-148a-3p

**Table 3:** Comparative analysis of pathways identified in this study versus the reported literature.

Pathway	This Study	Reported Literature
Adipogenesis	✓	[27,28]
Apoptosis	✓	[29]
Cardiac Electrophysiology	✓	[30,31]
Cardiac Muscle Contraction	✓	[10,12]
Cardiovascular System Development		[20]
Cell–Cell Adhesion	✓	[19,20,22]
Chromatin Organization		[22]
Circulatory System Development		[20]
EMT Process	✓	[32]
ER Stress	✓	[25]
Extracellular Matrix	✓	[19,20]
Inflammation	✓	[19,20]
Lipid and Atherosclerosis	✓	[19]
Lipid Metabolism	✓	[33]
Mitochondrial Respiration	✓	[22]
Oxidative Stress	✓	[23]
Oxidized LDL-Dependent Pathway		[23]
Platelet Degranulation		[19]
Regulation of Protein Secretion		[22]
Retinoic Acid Metabolic Process	✓	[34]
TGF-ß Signalling	✓	[35]
Tissue Development		[20]

## Data Availability

The RNA-seq data generated in this manuscript have been deposited in the NCBI Gene Expression Omnibus (GEO) under accession codes GSE273295 and GSE273298.

## Supplementary Materials

The following supporting information can be downloaded at https://www.mdpi.com/article/10.3390/biomedicines12081807/s1: Table S1: Raw counts of top 60 miRNAs; Table S2: list of miRNA (down)–gene (up) pairs; Table S3: list of miRNA (up)–gene (down) pairs.

## Abbreviations

ALDH1	Aldehyde dehydrogenase 1 family
ACM	Arrhythmogenic cardiomyopathy
BMPs	Bone morphogenetic proteins
FN1	Fibronectin
ITGA5	Integrin alpha 5
LP	Likely pathogenic
miRNAs	MicroRNAs
P	Pathogenic
RV	Right ventricle
SCD	Sudden cardiac death
VUS	Variant of unknown significance
