# Supplementary material for: Role of miRNA–mRNA Interactome in Pathophysiology of Arrhythmogenic Cardiomyopathy
Source: Biomedicines. 2024 Aug 9;12(8):1807. doi: 10.3390/biomedicines12081807 (PMC11351583; doi:10.3390/biomedicines12081807)
Supplement: Supplementary file 1 [file biomedicines-12-01807-s001.zip › Table S1.pdf]

Counts							
A1R	A2R	A4R	A6R	B1R	B2R	B5R	Gene_symbol
268147	333296	319027	39769	149975	223451	826406	hsa-miR-1-3p
110587	91623	77600	49265	38476	47353	180699	hsa-miR-143-3p
26647	70761	71180	51234	63687	81431	160503	hsa-let-7a-5p
27074	45652	39339	18103	25298	41027	111000	hsa-let-7f-5p
25882	52083	43790	25771	21360	25545	85002	hsa-miR-26a-5p
28171	45954	34588	8582	17237	12243	56518	hsa-miR-30d-5p
13406	53514	23570	6424	8590	6284	34382	hsa-miR-133a-3p
17567	26356	25662	9669	11579	12041	39905	hsa-miR-24-3p
17871	16799	14542	4624	5552	4409	25811	hsa-miR-30a-5p
10120	14886	12774	4398	5066	5808	20783	hsa-miR-126-3p
8811	13206	12865	4841	5804	6031	20016	hsa-miR-3074-5p
4907	18329	10663	3449	6000	6092	18876	hsa-miR-30c-5p
4371	12644	10991	5905	5633	5314	18177	hsa-miR-125b-5p
4524	11010	10114	5501	6172	6933	18603	hsa-let-7g-5p
6351	9710	7213	3066	3287	5284	15622	hsa-miR-27b-3p
2106	7043	6079	2008	5542	7547	16297	hsa-miR-125a-5p
3642	10930	6973	2556	3627	4263	12207	hsa-miR-23b-3p
4042	10743	6644	1492	2689	2598	10319	hsa-miR-378a-3p
2029	6048	4382	2687	4514	3126	9480	hsa-miR-92a-3p
2838	7362	5436	3769	2454	2178	6331	hsa-miR-23a-3p
3568	4394	3894	2565	3678	2126	9118	hsa-miR-16-5p
5040	4232	4240	1244	2229	2111	8984	hsa-miR-22-3p
2482	5222	4175	2261	1938	3021	8180	hsa-miR-26b-5p
2953	4191	2876	2977	4368	1119	7761	hsa-miR-451a
1496	3889	2983	1273	2556	2826	10222	hsa-miR-486-5p
1496	3889	2983	1273	2556	2826	10222	hsa-miR-486-3p
1872	3873	2903	2441	2029	2386	8070	hsa-let-7i-5p
1796	4135	3928	3380	1426	1829	7013	hsa-miR-199a-3p
817	2593	2470	3058	2414	3316	6456	hsa-let-7c-5p
3914	3111	3522	2173	1043	1457	5324	hsa-miR-21-5p
1840	3173	2807	1327	1658	1086	4991	hsa-miR-103a-3p
1840	3173	2807	1327	1658	1086	4991	hsa-miR-103b
2528	2397	2317	272	1096	1841	6253	hsa-miR-499b-3p
2528	2397	2317	272	1096	1840	6253	hsa-miR-499a-5p
1664	3486	2368	1272	1048	1185	5165	hsa-miR-99b-5p
1518	3079	2508	2021	848	946	4498	hsa-miR-99a-5p
2306	2956	2142	874	1256	1034	4838	hsa-miR-181a-5p
764	2217	1593	2737	1488	1875	4178	hsa-let-7b-5p
3068	2358	2571	523	976	664	4526	hsa-miR-30e-5p
1377	3945	2391	2752	671	508	2690	hsa-miR-145-5p
894	2055	1956	1684	712	909	3489	hsa-miR-199b-3p
1378	2869	1863	519	888	720	3044	hsa-miR-30e-3p
927	1861	1642	873	897	963	3769	hsa-miR-423-3p

927	1861	1642	873	897	963	3769	hsa-miR-3184-5p
912	1552	1243	2236	719	516	2638	hsa-miR-100-5p
951	2629	1462	1280	536	278	1718	hsa-miR-140-3p
1289	1841	1288	706	619	593	1990	hsa-miR-27a-3p
928	1654	1220	515	378	367	1917	hsa-miR-151a-3p
729	1841	1080	386	513	522	1785	hsa-miR-30a-3p
1095	1195	1101	748	560	487	1617	hsa-miR-29a-3p
913	1935	1215	466	387	249	1449	hsa-miR-30b-5p
603	1136	945	598	764	562	1959	hsa-miR-191-5p
267	588	593	469	840	1560	2217	hsa-let-7e-5p
543	1081	910	461	539	605	1712	hsa-miR-151a-5p
71	203	353	759	862	416	2886	hsa-miR-10a-5p
267	768	737	486	849	777	1490	hsa-let-7d-5p
431	821	735	377	481	704	1800	hsa-miR-98-5p
96	163	236	1239	872	287	1942	hsa-miR-10b-5p
495	983	831	383	366	320	1333	hsa-miR-148a-3p

Table S1. Raw counts of top 60 miRNAs.
